# Niche-Specific Adaptive Evolution of *Lactobacillus plantarum* Strains Isolated From Human Feces and Paocai

**DOI:** 10.3389/fcimb.2020.615876

**Published:** 2021-01-07

**Authors:** Qiqi Pan, Shi Cen, Leilei Yu, Fengwei Tian, Jianxin Zhao, Hao Zhang, Wei Chen, Qixiao Zhai

**Affiliations:** ^1^ State Key Laboratory of Food Science and Technology, Jiangnan University, Wuxi, China; ^2^ School of Food Science and Technology, Jiangnan University, Wuxi, China; ^3^ National Engineering Research Center for Functional Food, Jiangnan University, Wuxi, China; ^4^ Beijing Innovation Center of Food Nutrition and Human Health, Beijing Technology and Business University (BTBU), Beijing, China

**Keywords:** comparative genomics, *Lactobacillus plantarum*, niche-specific adaptive evolution, microbiome interaction, host physiology

## Abstract

*Lactobacillus plantarum*, a widely used probiotic in the food industry, exists in diverse habitats, which has led to its niche-specific genetic evolution. However, the relationship between this type of genetic evolution and the bacterial phenotype remains unclear. Here, six *L. plantarum* strains derived from paocai and human feces were analyzed at the genomic and phenotypic levels to investigate the features of adaptive evolution in different habitats. A comparative genomic analysis showed that 93 metabolism-related genes underwent structural variations (SVs) during adaptive evolution, including genes responsible for carbohydrate, lipid, amino acid, inorganic ion and coenzyme transport and metabolism, and energy production and conversion. Notably, seven virulence factor-related genes in strains from both habitats showed SVs — similar to the pattern found in the orthologous virulence genes of pathogenic bacteria shared similar niches, suggesting the possibility of horizontal gene transfer. These genomic variations further influenced the metabolic abilities of strains and their interactions with the commensal microbiota in the host intestine. Compared with the strains from feces, those from paocai exhibited a shorter stagnation period and a higher growth rate in a diluted paocai solution because of variations in functional genes. In addition, opposite correlations were identified between the relative abundances of *L. plantarum* strains and the genus *Bifidobacterium* in two media inoculated with strains from the two habitats. Overall, our findings revealed that the niche-specific genetic evolution of *L. plantarum* strains is associated with their fermentation abilities and physiological functions in host gut health. This knowledge can help guiding the exploration and application of probiotics from the specific niches-based probiotic exploitation.

## Introduction

*Lactobacillus* is a genus of gram-positive acid-tolerant bacteria that occupy a wide variety of niches. It is one of the most widely used probiotic species in the food industry because of its well-known health benefits to the host (Seddik et al., 2017). *L. plantarum* is a nomadic species that exists in a variety of habitats, including plants, the gastro-intestinal tracts of animals (including humans), and food materials (Martino et al., 2016).

Several studies have shown that the habitat diversity of probiotics leads to their niche-specific genetic evolution through single nucleotide polymorphisms (SNPs) and genomic structural variations (SVs) (Zhang, 2008; Barrick et al., 2009). The latter can be further classified into copy number variations caused by deletions, insertions or duplications; changes in orientation caused by inversions; and changes in chromosomal location caused by translocations and fusions (Wellenreuther et al., 2019). A previous study of a Western population identified 16 point mutations in the genome of the gut commensal microbe *Bacteroides fragilis* due to changes in the intestinal environment caused by dietary variations (Zhao et al., 2019). A comparative genomic study on 21 *L. salivarius* strains isolated from different habitats identified 15 genes that were involved in adaptation to the host, most of which encoded extracellular proteins and orthologs related to exopolysaccharide production (Lee et al., 2017). Another study on *L. mucosae* LM1 identified potential niche-specific genes for involved in the adaptation to various digestive tracts, such as glycogen metabolism- and folate biosynthesis- related genes. (Valeriano et al., 2019).

It should be noted that the characteristics of niche-specific evolution in *L. plantarum* genomes are controversial. A previous research based on phylogenomic and functional divergence analyses revealed that genomic variations in L. plantarum were uncoupled from their environmental origin (Martino et al., 2016) (Martino et al., 2016). Another study, which compared the genomes of 108 *L. plantarum* strains, showed that strain-specific genomic profiles were not associated with their original habitats (Sukjung et al., 2018). However, other studies reported that *L. plantarum* could undergo genomic variations because of ecological constraints, as indicated by the tendency of strains from the same habitats possess similar functional genomic profiles (Papizadeh et al., 2017; Cen et al., 2020). Also, a clinical study revealed that *L. plantarum* P-8 underwent genomic variations under the adaptive pressure of the host intestinal environment (Song et al., 2018). The differences between these experimental results were probably caused by diverse experimental and analytical methods and variations in the number of strains analyzed. Therefore, more studies are warranted to clarify the characteristics of niche-specific *L. plantarum* evolution.

Variations in bacterial genes, especially functional protein-encoding genes, are known to influence the bacterial phenotype. One study showed that different abundances of sugar-converting genes in three *L. paracasei* strains led to strain-level variations in carbohydrate utilization (Stefanovic and McAuliffe, 2018). Another study on the vaginal probiotic strain *L. rhamnosus* identified between-strain differences in genes related to the carbohydrate metabolic pathway and confirmed the phenotypic variations in a sugar utilization assay (Petrova et al., 2018).

Because of the wide usage of *Lactobacillus* species in food products, clarification of the relationship between the niche-specific adaptive evolution and phenotype of *Lactobacillus* spp. will be helpful in the development of potential probiotics. However, the majority of published studies in this area only have focused only on genome level changes, without further validation at the phenotypic level (Martino et al., 2016; Kelleher et al., 2017; Valeriano et al., 2019). Our previous research uncovered genomic variations in 140 *L. plantarum* strains (Cen et al., 2020). Nevertheless, the relationships between the genomic differences, original habitats, and phenotypes of *L. plantarum* strains remain unclear.

Therefore, in this study, a comparative genomic analysis was performed to identify niche-specific genomic variations in *L. plantarum* strains under adaptive pressure and associated these with various phenotypes. A fermentation assay was performed to verify the influences of adaptive evolution on the metabolism of these strains. In addition, 16S rRNA gene sequencing was performed to identify the correlation between the genomic variations in strains and the interaction of these strains with the host gut flora.

## Materials and Methods

### Bacterial Strains and Incubation Conditions

According to our previous research, six *L. plantarum* strains with different phylogeny were used in this study (Cen et al., 2020). Information about the six *L. plantarum* strains is listed in **Table 1**. The strains that had been preserved at - 80 °C in glycerol stock were grown overnight in de Man-Rogosa-Sharpe (MRS) broth (Sangon Biotech, Shanghai, China) at 37°C in an anaerobic workstation [the gas supply system was described in a previous article (Cen et al., 2020)], followed by 2% (v/v) subculture in fresh MRS.

**Table 1:** Information of six *L. plantarum* strains used in this study.

Strain	Region (District/City)	Origin	Accession No.	Reference
FCQNA23M1	Nanan, Chongqing	Human feces	SRR12559883	Cen et al., 2020
FCQNA28M4	Nanan, Chongqing	Human feces	SRR12559881	Cen et al., 2020
FCQNA29M3	Nanan, Chongqing	Human feces	SRR12559880	Cen et al., 2020
VCQWS1M2	Wansheng, Chongqing	Chinese paocai	SRR12559736	Cen et al., 2020
VCQLP6M2	Liangping, Chongqing	Chinese paocai	SRR12559737	Cen et al., 2020
VCQYB1M3	Yubei, Chongqing	Chinese paocai	SRR12559735	Cen et al., 2020

### Comparative Genomic Analysis

The whole-genome sequencing protocol and assembly and prediction methods were performed as described in a previous study (Zhai et al., 2019).

Orthologs in the six strains were defined using OrthoMCL (https://orthomcl.org/orthomcl/) with its default parameters (Fischer et al., 2011). Next, the chosen core-gene sequences were aligned using MAFFT-7.427 (https://mafft.cbrc.jp/alignment/software) (Katoh and Standley, 2013), and the phylogeny was inferred by the neighbor-joining method (Saitou and Nei, 1987). The phylogenetic tree was visualized using Evolview v2 online (www.evolgenius.info/evolview).

Orthologous proteins, carbohydrate-active enzymes, virulence factors, and antibiotic factors were annotated against the COG (Tatusov et al., 2000), CAZyme (Cantarel et al., 2009), VFDB (Chen et al., 2005) and CARD (Jia et al., 2017) databases, respectively, using BLAST (https://github.com/mosuka/blast) with its default parameters (Altschul et al., 1990).

### Growth of *Lactobacillus* Strains in a Paocai Diluent and Simulated Intestinal Juice

After sub-culturing twice, a 5% (v/v) inoculum of each of the six strains was inoculated into a paocai solution diluted with five parts of water (hereafter, the paocai diluent) and simulated intestinal juice, which was prepared according to a published method with minor modification (Minekus et al., 1999). Briefly, the fermentation medium was prepared as described in a previous study (Lau et al., 2016). Feces were washed twice with PBS and were dissolved with PBS to prepare the feces suspension in the ratio of 1:1 by weight. The feces suspension and the medium were mixed in the ratio of 10:1 by volume. The mixtures were incubated at 37°C in an anaerobic workstation.

Colony-forming units (CFUs) of all of the six strains grown in a paocai diluent were counted every 4 h during a 36-h period by the plate count method. The growth profiles of the strains in the simulated intestinal juice were measured by RT-PCR at 0, 6, 12, 24, and 36 h after inoculation. The design of the primer sequences and calculation of relative quantification were performed as described previously (Klocke et al., 2006). All experiments were conducted in triplicate.

### DNA Extraction and 16S rRNA Gene Sequencing

Cultures grown in the simulated intestinal juice were collected at the same time points as indicated for the RT-PCR assay. Metagenomic DNA was extracted using the FastDNA Spin Kit for Soil (MP Biomedicals, Shanghai, China), according to the manufacturer’s instructions. The V3-V4 regions of the bacterial 16S rRNA gene were amplified *via* PCR. The PCR primers and reaction program were as described previously (Fang et al., 2020). PCR products were gel-purified using GeneClean Turbo (MP Biomedicals, Shanghai, China). The DNA concentrations were measured using a dsDNA assay with NanoDrop spectrophotometers (Thermo, Shanghai, China). Subsequently, 16S rRNA sequencing was performed as described previously (Zhai et al., 2019).

### Sequence Processing and Bioinformatics Analysis

After sequencing, the 16S rRNA sequence data were analyzed using the QIIME2 (https://github.com/qiime2/) pipeline according to a previously published method (Zhai et al., 2019). Briefly, the raw sequences were aligned to exclude low-quality and short-length sequences. High-quality sequences with similarities > 97% were clustered into the same OTUs and representative sequences of each cluster were used to identify bacterial taxa. Beta diversity metrics were computed according to the Manhattan method in R-4.0.2 (https://cran.r-project.org/bin/windows/base/) using genus-level data.

### Statistical Analysis

All statistical analyses were performed using R-4.0.2. The pheatmap package was used to perform a cluster analysis of different functional genes between the two niche groups and to visualize. A principal component analysis (PCA) was performed using the prcomp function in the basic package, and the result was visualized using the ggbiplot package. The pairwise.t.test function was used to perform pairwise comparisons between the groups, and the wilcox.test function was used to perform two-sample Wilcoxon tests. A Pearson’s correlation analysis and a false discovery rate (FDR) were conducted using the psych package, and the correlation matrix was visualized using the corrplot package. A linear multiple regression model was established using the lm function in R, and its accuracy was tested using Spearman’s correlation (Johnson et al., 2019). The model was as follows: *r_n,i_* = *f* (*R_n_*_−1_, *G_j_*)where *r_n,i_* represents the relative abundance of the genus *i* at n hours after the strain was inoculated into the medium, *R_n-1_* represents the projection of the relative abundance of the microbiome on the PC1 and PC2 of PCA, and *G_j_* represents the projection of the inoculated strain’s functional gene numbers on the PC1 and PC2 of PCA.

## Results

### Functional Genomic SVs in *L. plantarum* Strains from Different Habitats

The six *L. plantarum* strains were divided into two groups based on their initial habitats (paocai-derived strains and feces-derived strains). Next, the core genes that differed between the two groups were selected and annotated using four databases to identify their functions.

**Figure 1:**
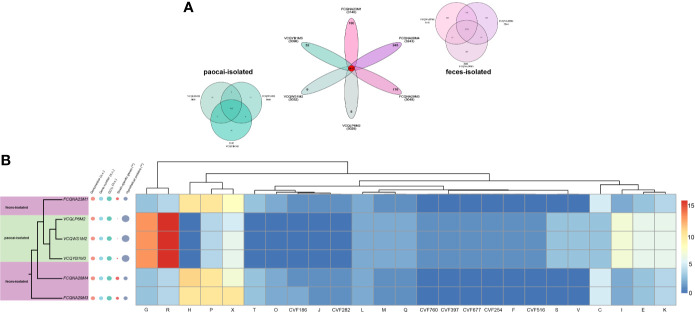
Comparative genomic and phylogenetic analyses of six *L. plantarum* strains. **(A)** Venn diagrams based on the pan genes of six *L. plantarum* strains isolated from paocai and human feces. **(B)** Phylogenetic tree of six *L. plantarum* strains constructed using the neighbor-joining method (left), and heatmap of the numbers of different core genes present in the strains’ genomes (the genes were annotated using four databases) (right). The basic information of strains’ genes, including genome sizes, gene numbers, GC%, and hypothetical protein numbers, is plotted beside the tree. The size of the circle represents the size of the value. n.s. represents p > 0.1, *represents p ≤ 0.1.

On average, 238 unique genes were identified in the human feces-derived strains, whereas only 33 unique genes were identified in the paocai-derived strains (**Figure 1A**). No significant differences (p > 0.05) were observed in the GC% and genome size between the two groups of strains (**Figure 1B**), indicating that natural selection in different niches led to SVs in only a few of the core genes. In particular, the mutation rates were 5.29% and 2.40% in the paocai-derived strains and feces-derived strains, respectively.

Functional annotations revealed the presence of SVs in 93 metabolism-related genes, including those involved in carbohydrate (G), lipid (I), amino acid (E), inorganic ion (P) and coenzyme (H) transport and metabolism, and energy production and conversion (C) (**Figure 1B**). Furthermore, 11 genes in the two groups classified into ‘mobilome, prophages, transposons’ category (X) were determined to have been acquired by horizontal gene transfer (HGT) (**Figure 1B**).

Notably, the annotation of virulence factors revealed that several virulence genes in the paocai-derived strains were orthologous to those of bacteria widely distributed in the soil and water (CVF254, CVF760, CVF677, and CVF197), whereas the virulence genes annotated in the human feces-derived strains were homologous to those of animal pathogenic bacteria (CVF282 and CVF516) (**Figure 1B**).

### Differences in the Growth Profiles of the Six *L. plantarum* Strains Between the Paocai Diluent and Simulated Intestinal Juice Media

To verify how genomic changes influence strains’ phenotypes, fermentation assays were conducted in a diluted paocai solution and simulated intestinal juice, which mimicked the initial habitats of the strains. A correlation analysis was then performed to associate the phenotypic variations with the genomic differences.

**Figure 2:**
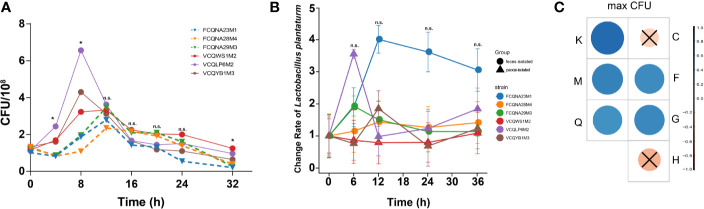
Growth profiles of *L. plantarum* strains in two media and their associations with genes. **(A)** Growth profiles of six *L. plantarum* strains in a paocai diluent. **(B)** Change rate of the relative abundance of six *L. plantarum* strains in the simulated intestinal juice. **(C)** Correlation grid plot for the correlation between maximum CFUs of *L. plantarum* strains in the simulated intestinal juice and functional genes. Red represents positive correlation; blue represents negative correlation. *represents p < 0.05; n.s. represents p > 0.05; × represents p > 0.05.

**Figure 2A** shows the growth profiles of the six strains in the paocai diluent. The paocai-derived strains had a shorter stagnation period and a higher growth rate, indicating that they could adapt to the fermentation broth environment more rapidly than the human feces-derived strains (p < 0.05). A further correlation analysis showed the maximum CFUs during growth were correlated with genes related to nucleotide (F) and carbohydrate transport/metabolism (G), secondary metabolites biosynthesis/transport/catabolism (Q), transcription (K) and cell wall/membrane/envelope biogenesis (M) (**Figure 2C**).

However, paocai- and human feces- derived *L. plantarum* strains did not exhibit differences in their rates of change in the relative abundance at any time point during growth in the simulated intestinal juice (p > 0.05, **Figure 2B**).

### Diverse Interactions Between *L. plantarum* Strains from Diverse Niches and the Host Commensal Bacteria

**Figure 3:**
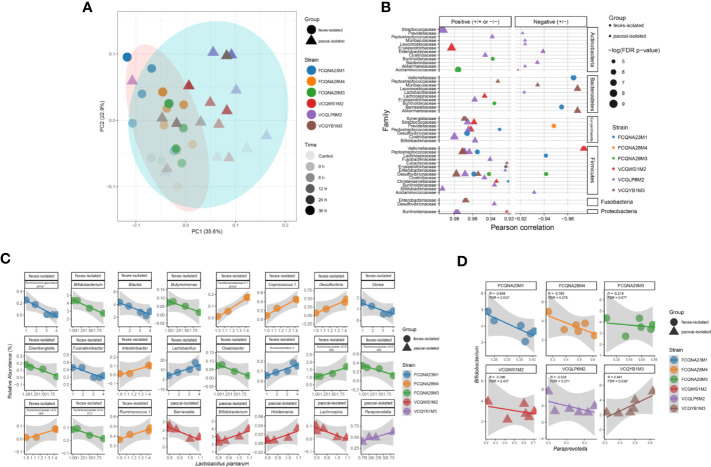
Diversity and correlation analyses between *L. plantarum* strains and microbiome in the simulated intestinal juice. **(A)** Variation of β diversity over time in the simulated intestinal juice. Correlation analysis between the relative abundance of each bacterial family **(B)** and between the genera *Bifidobacterium* and *Paraprevotella* **(D)** with the inoculation of different *L. plantarum* strains in the simulated intestinal juice. X and Y axes represent the relative abundances of the labelled genera. **(C)** Correlation analysis between *L. plantarum* strains and other genera in the simulated intestinal juice.

16S rRNA gene sequencing was performed to reveal the changes in the fecal microbiota structure in the simulated intestinal juice. The result showed that the strains from the two habitats had different regulatory effects on the intestinal flora over time (**Figure 3A**).

The Pearson’s correlation analysis of the relative abundances of gut microbiota at the family level showed that 44 couples of families were associated with bacterial family from different phyla in the medium inoculated with paocai-isolated strains, whereas in the medium inoculated with feces-isolated strains, only 13 families were demonstrated correlation with other families (FDR < 0.05) (**Figure 3B**). In particular, the medium inoculated with *L. plantarum* FCQNA28M4 (isolated from human feces), showed only one negative correlation between the Prevotellaceae family and one of the families in the Euryarchaeota phylum (R < 0, FDR < 0.05). In the medium inoculated with *L. plantarum* VCQLP6M2 (isolated from paocai), 26 couples of families were correlated with bacterial families belonging to different phyla (R > 0, FDR < 0.05).

Regarding the associations between *L. plantarum* and intestinal flora at the genus level, paocai- and feces- derived *L. plantarum* strains were correlated with five and 19 genera of the intestinal flora, respectively (FDR < 0.05). Among the 19 genera, 11 were negatively correlated with *L. plantarum* strains. In particular, *L. plantarum* VCQLP6M2 showed no significant association with any other bacterial genus (**Figure 3C**). Notably, the correlation between the relative abundances of *L. plantarum* FCQNA29M3 and *Bifidobacterium* was negative (R < 0, FDR < 0.05), whereas the correlation between the relative abundances of *L. plantarum* VCQWS1M2 and *Bifidobacterium* was positive (R > 0, FDR < 0.05) (**Figure 3C**).

Interestingly, *L. plantarum* strains in the two groups exhibited opposite influences on the associations between the *Bifidobacterium* and *Paraprevotella* genera (**Figure 3D**), such that the correlation between the relative abundances of these two genera was negative under the influence of the feces-isolated strain FCQNA23M1 (R < 0, FDR < 0.05), but was positive under the influence of the paocai-isolated strain VCQYB1M3 (R > 0, FDR < 0.05) (**Figure 3D**). These findings suggest that the adaptative evolution of strains in different niches plays a significant role in their interactions with the host gut flora.

### Relationship Between the Functional Genes of *L. plantarum* and the Commensal Gut Microbiota

To explore whether the diverse effects of *L. plantarum* strains on the intestinal flora are related to their genomes, linear multiple regression models with different functional genes as variables were established in this study.

**Figure 4:**
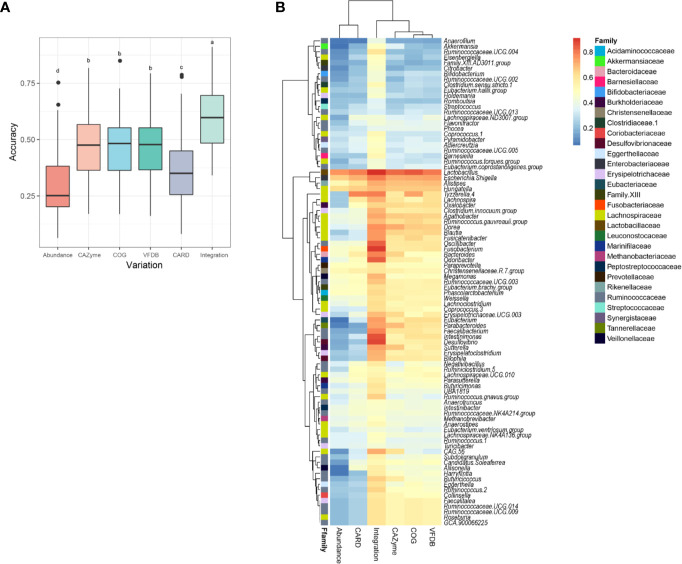
Correlation analysis between *L. plantarum* functional genomes and their influences on intestinal flora. **(A)** Boxplot for the accuracy scores of predicting the variation in microbiome using different functional genes. **(B)** Heatmap for the accuracy scores of predicting the variation in each genus using different functional genes. Labels of a, b, c, and d in the boxplot represent significant differences at p < 0.05.

The model in which the relative abundances of the microbiome were set as variables exhibited a low accuracy (**Figure 4A**). The introduction of functional gene annotations (from the four databases) as variables significantly improved the prediction accuracy of the model (p < 0.05, **Figure 4A**). This result suggests that the influences of the introduced strains on the intestinal flora were significantly related to the functional genes of the strains.

The prediction accuracy of the model for each genus in the gut community is presented in a heatmap (**Figure 4B**). Different correlation coefficients exist between various genera of the host gut microbiome and functional genes of *L. plantarum*. The majority of bacteria belonging to the Lachnospiraceae family exhibited strong correlations with the *L. plantarum* genome (average R > 0.5). However, several genera, including *Akkermansia* (R = 0.486), *Adlercreutzia* (R = 0.475), and *Holdemania* (R = 0.458), were only weakly associated with the functional genes of *L. plantarum*. These results indicate that a supplement of *L. plantarum* can interact with the intestinal bacteria *via* multiple pathways due to the adaptive evolution of its diverse functional genes.

## Discussion

Evolution involves sorting and variation. The former process involves two mechanisms, namely genetic drift and natural selection (Arber, 2000; Barrick et al., 2009; Gibson and Eyre-Walker, 2019). Natural selection can be further divided into positive selection, which promotes the spread of beneficial alleles, and negative selection, which hinders the spread of deleterious alleles (Barrick et al., 2009). These selections have strong influences on the phenotypes of an organism and should be taken into consideration when exploring and applying probiotics from various habitats.

In our previous study, 20 *L. plantarum* strains were isolated from different habitats, and their genomic variations in response to ecological constraints were identified by comparative genomics (Cen et al., 2020). However, the relationship between these genomic variations and the phenotypes remained unclear. Hence, in this study, six *L. plantarum* strains isolated from human feces and paocai were analyzed at the genomic and phenotypic levels to investigate the features of adaptive evolution in different habitats.

Previous studies have shown that only a few bacterial genes undergo SVs due to adaptive evolution, suggesting that evolution at the strain level is dominated by genetic drift, which occurs at a relatively slow rate (Gibson and Eyre-Walker, 2019; Vatanen et al., 2019). This pattern is consistent with our finding that strains isolated from the two different habitats had similar genome sizes and GC% (**Figure 1B**). Previous studies have also reported that differences in the abundances of functional genes can have significant effects on the phenotypes (Maldonado-Gómez et al., 2016; Hughes et al., 2020), and this is supported by our results (**Figure 1B**, **Figures 2A, C**). As the original habitats seem to play an important role in the variations of metabolism-related genes, this factor should be considered when exploring commercial probiotic strains.

Notably, the analysis of virulence factors in the two groups showed that several virulence genes in the paocai-derived strains were orthologous to those of bacteria widely found in soil and water, including *katA* of *Pseudomonas aeruginosa* (Chung et al., 2016), *Staphylococcus aureus* (Reiß et al., 2012) and *Yersinia enterocolitica* (Reuter et al., 2014), *gtcA* of *Listeria monocytogenes* (Promadej et al., 1999), *fbpC* of *Escherichia coli* (Blum et al., 2012) and *Vibrio antiquaries* (Hasan et al., 2015), and *agrA* of *Staphylococcus aureus* (Srivastava et al., 2014). However, virulence genes in the feces-derived strains were homologous to those of animal pathogenic bacteria, such as *rmlC* of *Salmonella enterica* (Graninger et al., 1999), *Bordetella bronchiseptica* (Park et al., 2012), *Streptococcus pneumoniae* (Ko et al., 2013), *Mycobacterium tuberculosis* (Norman et al., 2019) and *Shigella boydii* (Liu et al., 2008), and *fagC* of *Corynebacterium pseudotuberculosis* (Billington et al., 2002), *Corynebacterium ulcerans* (Trost et al., 2011), *Corynebacterium kutscheri* (Turnbull et al., 2019) and *Corynebacterium cystitidis* (Turnbull et al., 2019) (**Figure 2B**). This suggests that strains can obtain the virulence genes from the surrounding micro-environments through HGT, similar to the process by which bacteria acquire antibiotic-resistant genes (San Millan et al., 2016). Further investigation is warranted to uncover the mechanism by which virulence factors are transferred between organisms and the rate at which this transfer occurs. Considering the potential HGT of virulence factors, it is necessary to re-evaluate the safety of commonly recognized probiotics (Saarela, 2019; Sotoudegan et al., 2019).

The traditional Chinese pickled vegetable paocai is produced by fermentation without sterilization. The high salinity and acidity (average pH is 3.86) of paocai result in a relatively simple ecological environment dominated by the bacterial genera *Lactobacillus*, *Pediococcus*, *Serratia*, *Stenotrophomonas*, and *Weissella* (Liu et al., 2019). As a result, bacterial growth in the relatively low-diversity environment was strongly influenced by the chemical environment rather than the interaction between bacteria (Cao et al., 2017). Therefore, the strains adaptive to this kind of chemical environment had growth advantage when incubated in the medium with similar environment. The result in our study was consistent with this pattern (**Figure 2A**). This result could be attributable to the differences in metabolic-functional genes between the two groups of *L. plantarum* strains, especially those involved in carbohydrate (G) and coenzyme (H) transport/metabolism (**Figure 1B**, **Figure 2C**).

However, our *in-vitro* fermentation assays did not demonstrate significant associations between strains’ growth abilities and the adaptive evolution-triggered gene variations of the species (**Figure 2B**). Compared with paocai, the human colon is a sophisticated microsystem involving various types of bacteria-host cross-talks, and a weakly alkaline chemical environment containing trillions of microorganisms and diverse immune niches (James et al., 2020). In such a complex environment, bacterial growth is governed not only by bacterial metabolic genes, but also by the complex interactions between probiotics and the intestinal flora. Numerous mechanisms such as bacterial competition and mutualism govern these complex interactions (Bauer et al., 2018). Thus, our finding suggests that when developing new probiotic products, both the survival rates of the probiotic strains throughout the digestive tract and their growth profiles in the gut environment should be considered (Pham and Mohajeri, 2018).

The 16S rRNA gene sequencing data showed the complex relationships among the endogenous host gut bacteria under the influence of exogenous probiotics (**Figures 3B–D**). The comparative genomic analysis and phenotypic evaluation indicated that these relationships were possibly attributable to the different amounts of metabolites produced by strains from diverse initial habitats. For example, a previous study revealed that 5 μg/ml of exopolysaccharides (EPSs) isolated from *Bifidobacterium longum* suppressed lipopolysaccharide-induced cell growth inhibition, whereas 80 μg/ml of EPSs inhibited the growth of seven species of foodborne pathogenic bacteria (Wu et al., 2010). Furthermore, the data showed that only a few genera were affected by the probiotics (**Figure 3C**), and that the relative abundances of the other genera changed possibly in response to the metabolic cascades of the gut microbiota, which were strongly affected by the intestinal microenvironment (Coyte and Rakoff-Nahoum, 2019; Zmora et al., 2019). Similar to our findings, a previous study indicated that supplementation with *L. plantarum* ZLP001 increased the abundance of butyrate-producing bacteria, which in turn improved the butyrate content in the intestinal environment, strengthened the epithelial defense functions, and modulated the gut microbiota. Whether gene variations due to niche-specific evolution affect gut microbial cross-talks directly or change the metabolic cascades of the gut microbiota by regulating the intestinal chemical environment remains unclear. Further studies are needed to identify the relationships between genomic variations due to adaptive evolution and their effects on gut microbial genera, as this information can assist the design of probiotic preparations for the modulation of specific gut microbial species.

Overall, our study identified niche-specific adaptive evolution in the *L. plantarum* genome and provided an insight into how the SVs in functional genes affected the fermentation abilities of *L. plantarum* strains and their interactions with commensal microbes. This finding provides a promising outlook regarding the mining of new probiotic niches.

## Data Availability Statement

The datasets presented in this study can be found in online repositories. The names of the repository/repositories and accession number(s) can be found here: https://www.ncbi.nlm.nih.gov/, SRR12559883; https://www.ncbi.nlm.nih.gov/, SRR12559881; https://www.ncbi.nlm.nih.gov/, SRR12559880; https://www.ncbi.nlm.nih.gov/, SRR12559736; https://www.ncbi.nlm.nih.gov/, SRR12559737; https://www.ncbi.nlm.nih.gov/, SRR12559735.

## Author Contributions

QP: Methodology, software, formal analysis, visualization, writing—original draft. SC: Methodology, software, formal analysis, visualization. LY, FT, JZ, HZ: Validation, investigation. QZ: Conceptualization, writing—review and editing, supervision, funding acquisition. WC: Project administration, funding acquisition. All authors contributed to the article and approved the submitted version.

## Funding

This work was supported by the National Natural Science Foundation of China Program [No. 31871773 and No. 31820103010]; Projects of Innovation and Development Pillar Program for Key Industries in Southern Xinjiang of Xinjiang Production and Construction Corps [2018DB002]; National First-Class Discipline Program of Food Science and Technology [JUFSTR20180102]; the BBSRC Newton Fund Joint Centre Award; and Collaborative Innovation Center of Food Safety and Quality Control in Jiangsu Province.

## Conflict of Interest

The authors declare that the research was conducted in the absence of any commercial or financial relationships that could be construed as a potential conflict of interest.
